# Genetic Diversity and Emerging Trends of *Mycoplasma synoviae* in China: Insights from a 2024 Nationwide MLST Study

**DOI:** 10.3390/microorganisms14010257

**Published:** 2026-01-22

**Authors:** Lu Tu, Xuesong Li, Yiming Liu, Xin Jin, Lijuan Yin, Xiaoling Wang, Qingfeng Zhou, Kai Wang, Youzhi Tang

**Affiliations:** 1 College of Veterinary Medicine, South China Agricultural University, Guangzhou 510642, China; bluebabyno.1@163.com (L.T.); lxsong1207@126.com (X.L.); 2 Yunfu Branch, Guangdong Laboratory for Lingnan Modern Agriculture, Xinxing, Yunfu 527400, China; liu168156084@163.com (Y.L.); j1895755889@163.com (X.J.); wsyinlj@163.com (L.Y.); zhqf1012@163.com (Q.Z.); 3 Yunfu Branch, Yantai Animal Disease Prevention and Control Center, Yantai 264000, China; xlwang1220@163.com

**Keywords:** *Mycoplasma synoviae*, multilocus sequence typing, genetic evolution, MIC

## Abstract

*Mycoplasma synoviae* (MS) is a major pathogen threatening China’s poultry industry, causing severe economic losses, and clarifying its epidemiology is pivotal for disease control and flock purification. In this study, a total of 3215 chicken samples collected from 643 broiler farms across 15 provinces in China in 2024 were analyzed. PCR detected 14% positivity (450 samples), and 18 isolates obtained from these positive samples (4.0% isolation rate). Multilocus sequence typing (MLST, 7 housekeeping genes) and neighbor-joining phylogenetic analysis (integrating 425 reference sequences from public databases) identified 13 distinct sequence types (STs), demonstrating considerable genetic diversity among circulating MS strains. Pathogenicity assessment of the five isolates revealed that the infected chickens exhibited varying degrees of infectious synovitis, while no respiratory signs were observed. In addition, antimicrobial susceptibility testing against 10 commonly used antibiotics was conducted on the 18 strains, providing urgently needed guidance for rational drug use in the clinical treatment of both breeder and broiler flocks. This large-scale epidemiological study yields crucial insights into the current prevalence and genetic diversity of MS in China and lays a scientific foundation for formulating targeted prevention strategies and optimizing management practices.

## 1. Introduction

*Mycoplasma synoviae* (MS) is an important poultry pathogen capable of inducing various clinical diseases in chickens and turkeys [1]. Understanding its origin and distribution patterns is crucial for developing effective strategies for the prevention, control and eradication [2]. The causative agent can be transmitted both vertically (from breeder flocks to their offspring) and horizontally (among individuals within or between flocks) [3]. Although MS infection typically does not result in high mortality rates, clinical manifestations such as synovitis, arthritis, and airsacculitis can lead to increased condemnation rates of broiler carcasses at slaughter. In laying hens, MS significantly reduces egg production, resulting in substantial economic losses [4]. Due to the considerable impact of MS on poultry production, there is growing interest among poultry health professionals in early diagnosis, effective control measures, and the establishment of MS-free breeder flocks [5,6,7,8]. While antibiotic treatment can mitigate the effects of MS infection on production performance, the emergence of antimicrobial resistance often limits the efficacy of such interventions [9,10]. Consequently, identifying the sources and transmission routes of MS remains a critical component in preventing its spread and controlling its impact on poultry operations.

Routine diagnostic approaches for MS include pathogen isolation, serological assays, antibody detection, conventional PCR and quantitative real-time PCR assay. These methods are essential tools for identifying infected flocks. However, they exhibit significant limitations in strain differentiation and tracing the origin of infections [6,11,12]. Molecular typing methods enable discrimination between MS strains at the subspecies level, facilitate source tracking, and support epidemiological investigations [13,14]. Currently, molecular typing approaches for MS include DNA fingerprinting, whole-genome sequencing, and multilocus sequence typing (MLST). Among these, MLST has emerged as the preferred method for MS typing [15], offering high sensitivity and specificity while being applicable directly to clinical samples.

MLST was originally developed for *Mycoplasma gallisepticum* and was adapted for MS by Mohamed El-Gazzar in 2017, with the typing scheme incorporating seven housekeeping genes and the variable *vlhA* locus [16]. By sequencing seven housekeeping gene fragments with the *vlhA* locus, MLST allows determination of genetic relationships among strains. Studies have reported concurrent circulation of multiple MS strain types within the same poultry operation across time and space, underscoring the critical importance of robust strain typing for effective MS control [17]. In this study, we investigated the epidemiology of MS across multiple provinces in China in 2024 to analyze infection trends and understand the prevalence, evolutionary relationships, and dominant strain types circulating in different regions. Furthermore, we evaluate resistance profiles and inform targeted antimicrobial strategies. These findings provide critical insights for guiding MS prevention and treatment protocols, thereby helping to mitigate economic losses associated with MS infection in both breeder and broiler operations.

## 2. Materials and Methods

### 2.1. Subsection

This study was approved by the Animal Care Committee of South China Agricultural University (approval ID: 2025f040). All study procedures and animal care activities were conducted per the recommendations in the Guide for the Care and Use of Laboratory Animals of the Ministry of Science and Technology of the People’s Republic of China.

### 2.2. Sample Collection

To investigate the epidemiology of MS in China in 2024, a total of 3215 clinical samples including hock joints, organs and lung tissues were collected from 643 small-scale broiler farms in 15 provinces (Guangdong, Guangxi, Hunan, Hubei, Yunnan, Henan, Hebei, Chongqing, Sichuan, Anhui, Jiangsu, Shandong, Zhejiang, Fujian and Guizhou). A purposive sampling strategy was used, where farms and symptomatic birds (approximately 5–10 birds per flock) were selected based on clinical suspicion, such as lameness or joint swelling. None of the sampled flocks were vaccinated against MS. During field collection, tissue specimens were obtained using sterile disposable instruments (scalpels and forceps) to minimize contamination. Each specimen was placed into a sterile centrifuge tube and immediately stored in a portable cooler with ice packs at approximately 4 °C. These samples were transported to the laboratory under a continuous cold chain within 24–48 h. Upon arrival, further processing for pathogen isolation and molecular analysis was performed on a laminar flow bench to prevent secondary contamination. For long-term preservation, samples were stored at −80 °C. All tissue samples were collected aseptically on a laminar flow bench to avoid contamination.

### 2.3. MS Isolation and Detection

Clinical samples were dissected aseptically in a laminar flow biosafety cabinet. The tissues were then transferred into modified MS liquid growth medium and pre-cultured in a shaking incubator at 37 °C with 5% CO2 for 2–3 h. Following pre-culture, the suspension was filtered through a 0.45 μm membrane into a sterile tube. An equal volume of fresh modified MS medium was added, and the culture was incubated statically at 37 °C for 5–7 days. Successful bacterial growth was indicated by a color change of the medium from red to orange-yellow, reflecting acid production. To purify the isolate, the cultured broth was streaked onto modified MS solid medium plates. After incubation, characteristic fried egg-like colonies were selected and re-inoculated into liquid medium. This process of liquid-to-solid subculturing was repeated three times to ensure axenic purification of the MS strain. Genomic DNA extracted from tissue samples was used for MS-specific real-time qPCR detection. The detection protocol was optimized based on the method described by Xiaona Wei [18]. The percentage of MS-positive samples among the 3215 collected tissues was subsequently calculated.

### 2.4. MLST Analysis and Evolution Analysis

Seven housekeeping genes (*adk*, *atpG*, *efp*, *gmk*, *nagC*, *ppa*, and *recA*) were amplified using primer sets retrieved from the PubMLST database [16,19]. PCR amplification was carried out using a 25 μL reaction mixture consisting of: 12.5 μL of premixed Taq polymerase master mix, 0.5 μL each of forward and reverse primers (10 μM), 2 μL of genomic DNA template, supplemented with sterile distilled water to a final volume of 25 μL. Amplified products were sequenced via Sanger sequencing assay. Sequence alignment and assembly were performed using pyseer (v2.1.0). Novel allele sequences not present in the PubMLST database were assigned new allele identifiers upon submission. Each isolate was characterized by a seven-locus allelic profile, which was used to determine its ST. These profiles were compared against 425 reference sequences available in the PubMLST database to assess genetic relatedness and evolutionary trends among the isolates [18]. All 425 MS reference strains are available in the PubMLST database, representing isolates from diverse geographical regions including the Americas, Europe, Australia, and Asia. For each strain, the sequences of the seven housekeeping genes were concatenated into a single sequence for downstream analysis. Multiple sequence alignment was performed using MAFFT (v7.525), and a phylogenetic tree was constructed with FastTree (v2.1) under the appropriate evolutionary model to infer genetic relationships among the global and newly isolated MS strains.

### 2.5. Animal Experiment Design

To evaluate the current pathogenicity status of circulating MS strains, five isolates (MS14, MS31, MS32, MS33, and MS34) were randomly selected from diverse sources among the most recent 2024 collection. A total of sixty 1-day-old SPF chicks were randomly divided into six groups (n = 10 per group) for the pathogenicity assessment. Five groups were inoculated intramuscularly in the left leg with 0.2 mL of 5 purified MS isolates. One additional group served as a negative control and received 0.2 mL of sterile modified Frey’s medium via the same route. All groups were housed in separate isolators to prevent cross-contamination. Clinical signs (including depression, ruffled feathers, and lameness), feed and water intake, and mortality were monitored and recorded daily throughout the 14-day experimental period. At the end of the 14-day experiment, the surviving chicks were euthanized and subjected to necropsy. Pathological changes in the internal organs were observed and recorded. In view of the joint-origin of the isolates and the absence of overt respiratory symptoms throughout the trial, pathological evaluations were focused on joint lesions to evaluate MS-induced synovitis. All statistical analyses were performed using GraphPad Prism 10.0. Pathological damage was assessed using a composite scoring system. Five specific sites were evaluated for each bird: the bilateral hock joints, bilateral footpads, and the sternal bursa. Each site was graded on a scale of 0 to 4 based on the severity of lesions, resulting in a maximum possible score of 20 per bird. The final composite score for each individual was calculated by summing the scores from all five observation sites. The Kruskal–Wallis H test was performed to compare the overall differences among groups and representative necropsy images were provided to illustrate the observed pathological changes.

### 2.6. Minimal Inhibitory Concentration

To evaluate the susceptibility of MS isolates to antimicrobial agents, a total of ten antibiotics, including tylosin (TY), doxycycline (DO), tiamulin (TIA), lincomycin (LIN), gentamicin (GM), tilmicosin (TIM), enrofloxacin (ENR), chlortetracycline (CTC), oxytetracycline (OTC), and sarafloxacin (SAL), were employed in this study to assess drug susceptibility. Each antibiotic was prepared at a stock concentration of 2048 μg/mL and sterilized by filtration through a 0.22 μm membrane. Minimum inhibitory concentration (MIC) assays were performed in 96-well microdilution plates according to the method described by Li et al. [20]. In brief, 100 μL of MS broth medium was aliquoted into each well. Then, 100 μL of the antibiotic solution was added to the first column and serially diluted two-fold across the plate, resulting in a final concentration range from 1024 μg/mL to 0.06 μg/mL in the test wells. An MS bacterial suspension adjusted to 10^5^ CCU/mL was inoculated into each well, leading to an additional two-fold dilution of the antibiotic concentrations. Each assay included a negative control (medium and inoculum without antibiotic) and a drug sterility control (medium and antibiotic without inoculum). The plates were incubated at 37 °C under appropriate atmospheric conditions until the color of the negative control well changed to orange-yellow, indicating bacterial growth. The MIC was defined as the lowest concentration of antibiotic that prevented this color change (MIC_100_, indicating 100% growth inhibition).

## 3. Results

### 3.1. Epidemiological Investigation of Clinical Samples

In this study, an epidemiology was performed across 15 provinces in China, including Guangdong, Guangxi, Hunan, Hubei, Yunnan, Henan, Hebei, Chongqing, Sichuan, Anhui, Jiangsu, Shandong, Zhejiang, Fujian, and Guizhou. The presence of MS was evaluated using real-time qPCR, which detected MS in 450 samples, corresponding to a positivity rate of 14.0% (450/3215). Regional variation in infection rates was observed, with Jiangsu showing the highest prevalence (28.33%) and Shandong the lowest (4.29%). Detailed provincial-level prevalence data are summarized in Table 1.

### 3.2. MS Isolation and MLST Analysis

Among the 450 MS-positive samples, 18 MS strains were successfully isolated, yielding an isolation rate of 4.0%. Total genomic DNA was extracted from each isolate, and both the *vlhA* gene and the seven housekeeping genes used for MLST analysis were amplified and sequenced to support subsequent phylogenetic and molecular evolutionary analyses. The allelic combinations and assigned sequence types (STs) are listed in Table 2.

The MLST analysis of seven housekeeping genes from 18 MS strains was conducted using pyseer (v2.1.0) following the standardized typing scheme available on the PubMLST website. The resulting MLST profiles are summarized in Table 2. Notably, the *recA* gene sequences from all 18 isolates and the *nagC* gene sequences from seven isolates did not match any existing entries in the PubMLST database, confirming that these represent novel sequence types (STs). This finding underscores the genetic diversity and ongoing evolution of MS strains circulating in China and highlights the importance of continuous molecular surveillance for tracking emerging variants.

**Figure 1:**
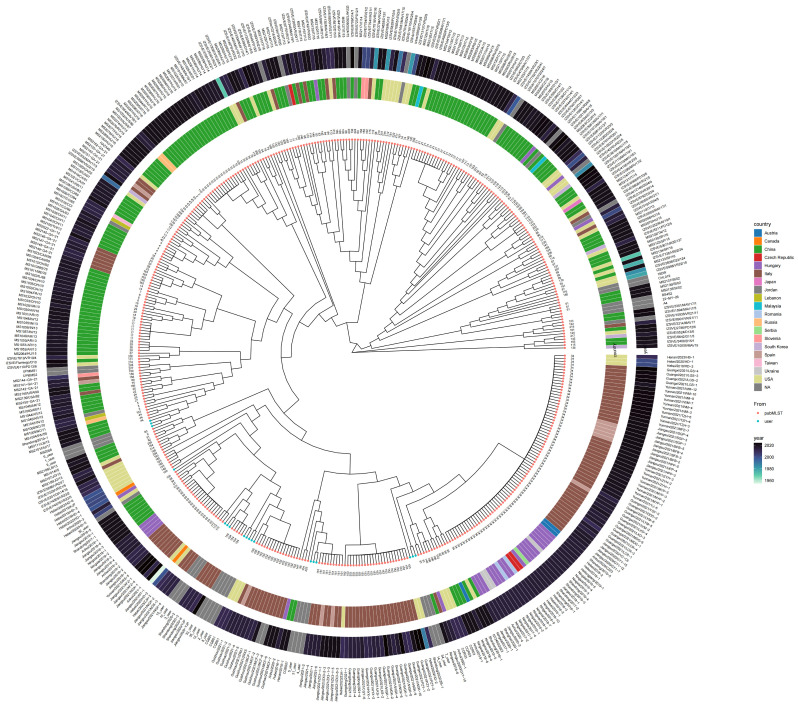
Phylogenetic analysis of MS isolates based on multilocus sequence typing (MLST). A neighbor-joining phylogenetic tree was constructed using concatenated sequences of seven housekeeping genes from 18 isolates (highlighted in blue) obtained in this study together with 425 reference strains retrieved from the PubMLST database. The innermost ring denotes strain identifiers, the middle ring represents sequence types (STs), and the outer ring indicates phylogenetic group classifications. The 18 isolates were distributed across multiple clades, some clustering with strains previously reported in Europe (e.g., Spain, Hungary, Italy). The presence of multiple novel STs highlights the substantial genetic diversity and ongoing evolution of *M. synoviae* circulating in China.

Concatenated sequences of the seven loci were analyzed together with 425 global reference strains to construct a phylogenetic tree (Figure 1). The 18 isolates were distributed across multiple clades. Notably, MS01, MS09, and MS06 formed a single clade, and MS03, MS10, and MS15 grouped into one distinct clade. MS32 was placed on a separate branch closely related to previously reported Chinese isolates. MS02, MS08, MS12, MS33, and MS35 formed another clade with close phylogenetic affinity to strains from Spain. MS07, MS14, and MS34 clustered together and showed genetic similarity to isolates from China, Spain, and Hungary. MS04, MS05, and MS31 formed an independent branch more closely related to strains originating from Italy.

### 3.3. Pathogenicity Analysis of MS Isolates

**Figure 2:**
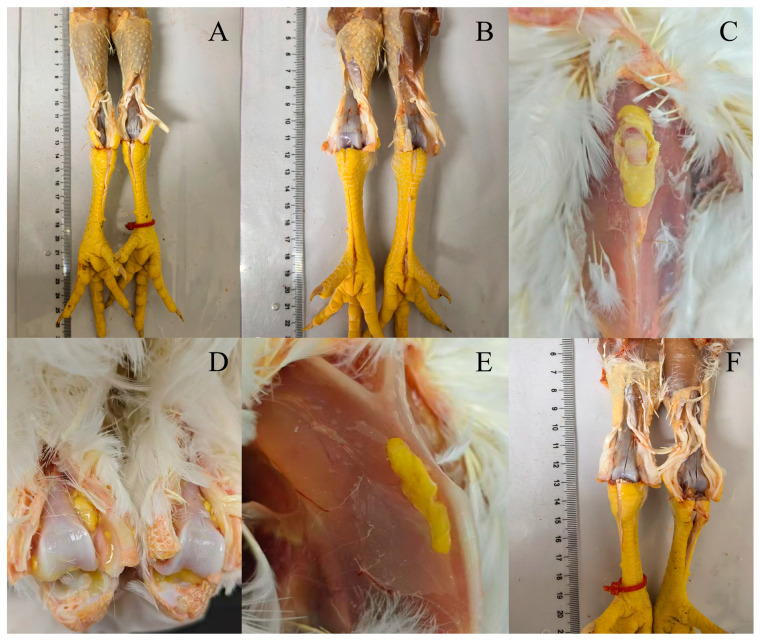
Gross lesions observed in SPF chickens following experimental infection with MS isolates. Representative necropsy images show strain-dependent variation in joint and thoracic lesions. (**A**,**B**) SPF chickens inoculated with strains MS14 (**A**) and MS31 (**B**) exhibited increased synovial fluid in the tarsal joint, with no caseous exudate present in the joint or sternal bursa. (**C**) MS32 infection resulted in prominent thoracic cyst-like lesions. (**D**) MS33 infection produced typical caseous exudate adhering to the tarsal joint. (**E**) MS34 infection led to thoracic cyst-like lesions similar to those observed with MS32. (**F**) Negative control birds showed no gross lesions.

**Figure 3:**
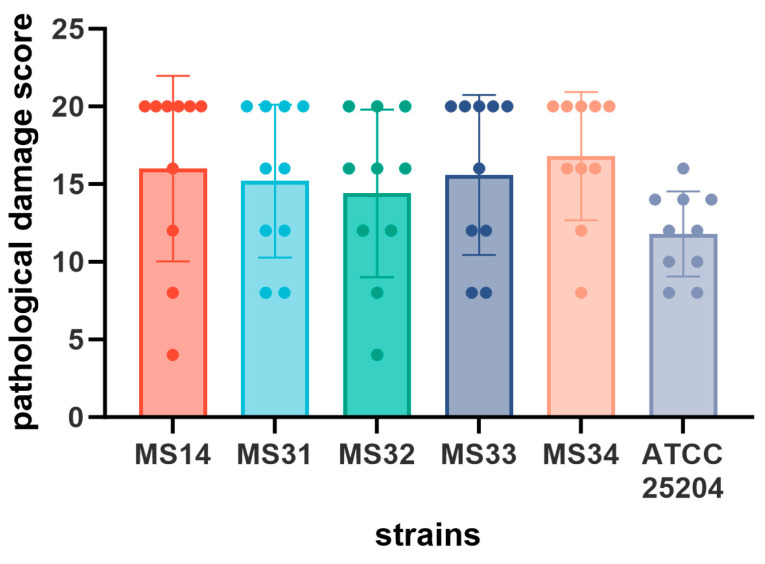
Composite pathological damage scores of the MS isolates and *M. synoviae* ATCC 25204 reference strain. The scores represent the cumulative pathological severity across five anatomical sites (max score = 20). Each bar represents the mean ± SD, and individual data points correspond to each chick (n = 10).

Five isolates (MS14, MS31, MS32, MS33, and MS34) representing distinct phylogenetic clades were selected for pathogenicity testing. SPF chickens inoculated with these strains exhibited varying degrees of clinical signs, including depression, reduced feed intake, and impaired leg movement over the 14-day observation period. While clinical parameters were monitored, body weight gain was not statistically analyzed because mortality in certain infected groups would have resulted in inconsistent and non-comparable datasets. Necropsy revealed typical lesions associated with MS infection, such as excessive clear mucus accumulation within tarsal joint cavities and, in advanced cases, yellow caseous exudate in joints and sternal bursae (Figure 2). To provide a quantitative evaluation, pathological damage was recorded using a composite scoring system (Figure 3). Despite the lack of statistical significance among the groups due to large variances, a visible trend was observed when compared to the reference strain. No respiratory lesions were observed in any infected birds during the 14-day observation period. Furthermore, MS was successfully re-isolated from the hock joints of both deceased and euthanized birds. Subsequent MLST analysis confirmed that the re-isolated strains were identical to the original challenge strains.

### 3.4. Drug Susceptibility Testing

To evaluate the susceptibility of MS isolates to antibiotics, antimicrobial susceptibility testing for all isolated MS strains is performed in this study. A concerning trend of increasing resistance to multiple antimicrobial agents was observed among the isolates (Table 3). All the MS strains demonstrated high susceptibility to DO, TY, and TIA, with MIC values ≤0.06 μg/mL. Variable susceptibility was observed for LIN, TIM, and CTC, with MIC values ranging from 0.25 to 64 μg/mL. Notably, elevated MIC values (>1 μg/mL) were consistently recorded for EN, GM, and SAL, indicating reduced efficacy of these agents. For OTC, MIC values exhibited considerable variability, spanning from 0.06 to 64 μg/mL across different strains. These findings underscore the importance of ongoing antimicrobial susceptibility surveillance and suggest that doxycycline, tyvalosin, and tiamulin remain the most reliably effective options among the tested antimicrobials for managing MS infections in the studied regions.

## 4. Discussion

*Mycoplasma synoviae* (MS) poses a significant threat to poultry production in China, causing leg abnormalities and respiratory disorders in breeders as well as reduced eggshell quality. In broilers, MS infection frequently leads to arthritis and airsacculitis, impairing growth performance and resulting in considerable economic losses. Regular surveillance and epidemiological investigation are essential for controlling MS transmission. In this study, 3215 samples were collected from 643 small-scale broiler farms across 15 Chinese provinces in 2024 to evaluate MS prevalence. Among these, 450 samples tested positive, yielding a positivity rate of 14.0%. Compared with earlier multi-province epidemiological studies by Sui et al. [21] and Wei et al. [14], the prevalence of MS in China appears to have decreased in 2024. Notably, this study adopted a purposive sampling approach targeting symptomatic flocks—a strategy known to overestimate prevalence relative to random surveillance [22]. What is striking here is that our detection rate (14.0%) still fell below previous reports, even accounting for this inherent sampling bias. This observation strongly points to a genuine downward trend in MS prevalence. We hypothesize this trend may stem from improved biosecurity awareness among small-scale poultry farmers, coupled with increased use of prophylactic antimicrobials. Beyond this, regional variations in farm density and management practices across the 15 provinces are likely to have shaped the observed epidemiological patterns. Specifically, higher farm density tends to facilitate rapid MS transmission by reducing the distance for pathogen spread, while inconsistencies in biosecurity implementation across small-scale operations influence the pathogen’s persistence in local poultry populations. Furthermore, a notable discrepancy was observed between the real-time qPCR positivity rate (14.0%) and the successful isolation rate (4.0%). This gap can be attributed to several limiting factors. First, the time elapsed between infection and sampling is critical, as MS is more readily isolated during the acute stage, whereas DNA remains detectable by qPCR even in chronic stages when viable bacterial loads have diminished [23]. Second, the history of prior antibiotic treatment on these small-scale farms likely suppressed the growth of viable MS, hindering culture efforts while not affecting molecular detection [24]. Finally, the fastidious nature of MS and the specific tissue types selected for culture may have further limited isolation success compared to the high sensitivity of real-time qPCR. Nevertheless, sustained surveillance and rational drug use remain critical to preventing resistance development and managing MS outbreaks effectively.

MLST is a well-established molecular method for bacterial and mycoplasma strain characterization, having been applied to numerous species including *Mycoplasma bovis* [25], *Mycoplasma pneumoniae* [26], *Mycoplasma hyopneumoniae* [27], *Mycoplasma genitalium* [28], *Mycoplasma urealyticum* [29], and *Mycoplasma gallisepticum* [18]. In this study, MLST analysis of the 18 isolated MS strains—based on seven housekeeping genes (*adk*, *atpG*, *efp*, *gmk*, *nagC*, *ppa*, and *recA*)—revealed 13 novel STs not previously documented in the PubMLST database. Notably, the *recA* gene contained a previously unreported allele, while *nagC* was found to carry a new allele variant present in the seven most genetically divergent strains. These findings indicate a significant shift in the genotypic landscape of MS circulating in China in 2024 compared to earlier domestic reports, highlighting ongoing evolution and possible adaptation under local epidemiological and selection pressures.

The PubMLST database—a publicly accessible online resource—hosts curated reference data contributed by researchers globally, supporting molecular epidemiological and evolutionary studies [19]. Phylogenetic analysis based on seven housekeeping genes from the MS isolates demonstrated close relationships with isolates originating from China, Spain, and Hungary. Notably, all isolates formed a clade phylogenetically distinct from the Australian attenuated vaccine strain MS-H, consistent with previously reported MS typing results in China. These findings highlight the considerable genetic variability and dynamic evolution of MS strains within Chinese poultry flocks [15]. The observed diversity of genotypes and their phylogenetic proximity to strains from distant geographical regions, such as Europe, suggests a complex evolutionary history for MS in China. The high degree of genetic relatedness observed in this study primarily underscores the widespread distribution of specific evolutionary lineages. However, further research incorporating animal movement and trade data is required to clarify the exact transmission routes. At present, our results emphasize the ongoing genetic diversification and the presence of globally-related lineages within local poultry populations.

Virulence evaluation of MS isolates revealed varying degrees of pathogenicity in SPF chickens, with clinical manifestations including depression, reduced feed intake, and lameness. Necropsy observations showed increased synovial fluid and mucus accumulation in joint cavities, with later stages characterized by caseous exudate in joints and sternal bursae. To provide a more objective and rigorous comparison, we implemented a composite pathological scoring system evaluating multiple anatomical sites. While numerical trends suggested that certain isolates, such as MS14, induced more pronounced damage, the differences among the infected groups did not reach statistical significance (*p* > 0.05). This lack of significance may be attributed to the high individual variability in host response and the relatively small sample size per group, which are common challenges in quantifying the subtle virulence variations of *Mycoplasma* species. Importantly, the successful re-isolation of MS from the hock joints and the confirmation of their genotypes *via* MLST fulfilled Koch’s postulates, verifying that the observed lesions were directly induced by the challenged strains and ensuring the reliability of our pathogenicity data. Notably, none of the MS-infected SPF chickens in this study exhibited respiratory signs throughout the experimental period. Consequently, air sac examinations were not included in the pathological evaluation, as our assessment was prioritized toward the manifested joint-related lesions. This may be due to the fact that all strains were isolated from tarsal joints, suggesting possible tissue tropism and route-specific adaptation. MS is frequently reported in mixed infections with other pathogens, contributing to severe economic losses across broiler, layer, and breeder operations. Comprehensive understanding of the virulence and epidemiological profiles of circulating MS strains is therefore essential for designing effective intervention strategies and vaccination programs [30,31]. These findings underscore the need for further investigation into genotype–phenotype correlations and tissue tropism among emerging MS lineages.

The inherent challenges in isolating viable MS, particularly following antimicrobial intervention, further underscore the importance of rational drug use in treating MS infections. As cultural isolation becomes considerably more difficult once treatment has begun, evidence-based antimicrobial stewardship remains a cornerstone of current disease control strategies. Through the antimicrobial susceptibility testing (AST) of our isolates, we demonstrated that such profiling can effectively guide clinical drug selection [9,32]. Our findings showed that all tested strains were susceptible to DO, TY, and TIA, which is different from a recent report by Zhang et al. [32] regarding the continued efficacy of pleuromutilins and tetracyclines against MS in China. In addition, the increasing trend in resistance observed among fluoroquinolones and macrolides mirrors the epidemiological shifts documented in previous studies, likely reflecting the long-term selective pressure of these drug classes in poultry production [33]. It is important to note that testing only 10 antimicrobial agents presents a limitation in the representativeness of the susceptibility profile. Future studies should include broader drug panels to better inform treatment protocols and antimicrobial stewardship in poultry production systems.

## 5. Conclusions

This study provides updated insights into the epidemiology, genetic diversity, and antimicrobial susceptibility of MS in China. The identification of numerous novel STs highlights the rapid evolution of MS and reinforces the importance of sustained molecular surveillance. Combined with regional prevalence data and antimicrobial susceptibility patterns, these findings offer a valuable scientific basis for developing targeted prevention and control strategies, including strain-specific monitoring, rational antimicrobial use, and improved biosecurity measures. Future work integrating whole-genome sequencing and longitudinal sampling will further elucidate the drivers of MS evolution and transmission dynamics in China.

## Figures and Tables

**Table 1:** Provincial distribution and positivity rates of MS in clinical samples collected in China during 2024.

Province	Sample Numbers	MS Infection	
		Positive Number	Positive Rate
Guangdong	465	104	22.37%
Guangxi	335	81	24.18%
Hunan	245	32	13.06%
Hubei	185	17	9.19%
Yunnan	50	6	12%
Henan	100	9	9%
Hebei	100	12	12%
Chongqing	100	14	14%
Sichuan	50	4	8%
Anhui	275	17	5.10%
Jiangsu	300	85	28.33%
Shandong	280	12	4.29%
Zhejiang	400	32	8.00%
Fujian	240	21	8.75%
Guizhou	90	4	4.44%
Total	3215	450	14%

**Table 2:** MLST allelic profiles and sequence types of 18 MS isolates collected in 2024.

Number	Strains	Sample	Region	*adk*	*atpG*	*efp*	*gmk*	*nagC*	*ppa*	*recA*	ST
1	MS01	Jiangsu/2024	Jiangsu	2	3	2	3	3	2	57	245
2	MS02	Guangdong/2024	Guangdong	4	38	2	1	46	19	46	246
3	MS03	Yunnan/2024	Yunnan	4	38	19	2	46	19	46	247
4	MS04	Guangxi/2024	Guangxi	34	38	19	1	10	19	56	248
5	MS05	Guangxi/2024	Guangxi	34	38	19	1	80	19	56	249
6	MS06	Jiangsu/2024	Jiangsu	2	3	2	3	81	2	57	250
7	MS07	Zhejiang/2024	Zhejiang	28	38	19	1	10	19	48	251
8	MS08	Guangdong/2024	Guangdong	6	6	4	1	82	19	59	252
9	MS09	Jiangsu/2024	Jiangsu	2	3	2	3	3	2	57	245
10	MS10	Sichuan/2024	Sichuan	4	38	19	2	46	19	46	247
11	MS12	Guangdong/2024	Guangdong	4	38	2	1	46	19	46	246
12	MS14	Zhejiang/2024	Zhejiang	28	38	19	1	76	19	48	256
13	MS15	Chongqing/2024	Chongqing	4	38	19	2	77	19	46	257
14	MS31	Guangxi/2024	Guangxi	34	38	19	1	10	19	56	248
15	MS32	Shandong/2024	Shandong	28	38	19	1	46	19	51	259
16	MS33	Guangdong/2024	Guangdong	4	38	2	1	78	19	46	260
17	MS34	Zhejiang/2024	Zhejiang	28	38	19	1	10	19	48	251
18	MS35	Hunan/2024	Hunan	4	38	2	1	79	19	46	262

**Table 3:** Minimum inhibitory concentrations (MICs) of 10 antimicrobial agents against 18 MS isolates.

Strains	MICs (μg/mL)									
	TY	DO	TIA	LIN	GM	TIM	EN	CTC	OTC	SAL
MS01	<0.03	<0.03	<0.03	16	2	16	2	0.25	1	4
MS02	<0.03	<0.03	<0.03	16	64	32	4	0.5	4	4
MS03	<0.03	<0.03	<0.03	32	4	64	8	1	2	128
MS04	<0.03	<0.03	<0.03	32	4	64	2	0.25	0.25	16
MS05	<0.03	<0.03	<0.03	16	8	64	2	32	0.5	64
MS06	<0.03	<0.03	<0.03	16	16	32	4	0.25	0.5	128
MS07	<0.03	<0.03	<0.03	32	16	32	4	2	8	128
MS08	<0.03	<0.03	<0.03	16	4	64	8	4	4	64
MS09	<0.03	<0.03	<0.03	64	4	32	4	4	4	32
MS10	<0.03	<0.03	<0.03	16	2	32	4	8	2	32
MS12	<0.03	<0.03	<0.03	32	32	64	4	16	0.25	4
MS14	<0.03	<0.03	<0.03	16	32	64	2	32	16	4
MS15	<0.03	<0.03	<0.03	32	64	64	16	64	16	4
MS31	<0.03	<0.03	<0.03	64	64	32	16	64	64	8
MS32	<0.03	<0.03	<0.03	64	32	32	8	32	64	2
MS33	<0.03	<0.03	<0.03	16	16	16	2	16	0.5	64
MS34	<0.03	<0.03	<0.03	32	32	32	4	0.25	1	2
MS35	<0.03	<0.03	<0.03	32	32	16	8	1	2	4

Abbreviations: TY, tyvalosin; DO, doxycycline; TIA, tiamulin; LIN, lincomycin; GM, gentamicin; TIM, tilmicosin; EN, enrofloxacin; CTC, chlortetracycline; OTC, oxytetracycline; SAL, sarafloxacin.

## Data Availability

The original contributions presented in this study are included in the article. Further inquiries can be directed to the corresponding author.

## Abbreviations

The following abbreviations are used in this manuscript:

CCU/mL	Color Changing Units per Milliliter
PCR	Polymerase Chain Reaction
